# A Pan-Cancer Analysis on the Systematic Correlation of MutS Homolog 2 (MSH2) to a Malignant Tumor

**DOI:** 10.1155/2022/9175402

**Published:** 2022-03-24

**Authors:** Hai Yao, Zhidong Cao, Haochuan Yong, Xiaoxing Zhang, Xin Zhang, Wei Li, Shenshen Zhi, Wenyan Wu

**Affiliations:** ^1^Department of Orthopaedic Surgery, Chongqing Emergency Medical Center, Chongqing University Center Hospital, School of Medicine, Chongqing University, Chongqing 400014, China; ^2^Department of Clinical Laboratory, Chongqing Emergency Medical Center, Chongqing University Center Hospital, School of Medicine, Chongqing University, Chongqing 400014, China

## Abstract

MutS homolog 2 (MSH2) is a crucial participant in human DNA repair, and lots of the studies functionally associated with it were begun with hereditary nonpolyposis colorectal cancer (HNPCC). MSH2 has also been reported to take part in the progresses of various tumors' formation. With the help of GTEx, CCLE, and TCGA pan-cancer databases, the analysis of MSH2 gene distribution in both tumor tissues and normal control tissues was carried out. Kaplan-Meyer survival plots and COX regression analysis were conducted for the assessment into the MSH2's impact on tumor patients' clinical prognosis. In an investigation to the association of MSH2 expression with immune infiltration level of various tumors and a similar study on tumor immune neoantigens, microsatellite instability was subsequently taken. It was found that high expression of MSH2 is prevalent in most cancers. MSH2's efficacy on clinical prognosis as well as immune infiltration in tumor patients revealed a fact that expression of MSH2 in prostate adenocarcinoma (PRAD), brain lower-grade glioma (LGG), breast-invasive carcinoma (BRCA), and head and neck squamous cell carcinoma (HNSC) posed a significant correlation with the immune cell infiltration level of patients. Likewise as above, MSH2's expression comes in a similar trend with tumor immune neoantigens and microsatellite instability. MSH2's expression in the majority of tumors is a direct factor to the activation of tumor-associated pathways as well as immune-associated pathways. MSH2's early screening or even therapeutic target role for sarcoma (SARC) diagnosis is contributing to the efficiency of early screening and overall survival in SARC patients.

## 1. Introduction

Protein MutS homolog 2 (MSH2, ENSG00000095002) is a component of DNA damage repair by guiding the generation of critical relevant protein. This protein helps repair errors arising when DNA is replicated for cell division proteins (the MSH2 protein binds to one of the MSH6 or MSH3 (each produced by a different gene)) to form a dimer of the two-protein complex [1], which recognizes the error-occurring sites on DNA that begets in the course of DNA replication. The MLH1-PMS2 dimer is formed with another set of proteins, which subsequently combine with the MSH2 dimer to initiate the process of error repair by removing mismatched DNAs and replicating a new fragment [2, 3]. DNA damage is an inducement of cancer genesis; hence, the defection of DNA repair genes is primarily responsible for many cancers' initiation and development [4, 5]. Methylation in a promoter might contribute to a decline in DNA repair via the 4 pathways where MSH2 is involved: the repair to DNA loss of match, transcription-coupled repair, homologous recombination, and the repair to base excision [6–8]. This reduction in repair capacity might bring forth accumulation of DNA damage and lead to carcinogenesis [9]. It was reported in hereditary nonpolyposis colorectal cancer (HNPCC) that 40% of the genetic variants are the disease-associated ones of MSH2 and they are the primary inducements of HNPCC development [10]. A study on the MSH2 in non-small-cell lung cancer (NSCLC) suggested that although the gene was not mutated, 29% of NSCLC cases were found with decline in epigenetic expression of MSH2 [11].

Likewise in the case of no MSH2 mutation found, MSH2 promoter methylation was found in 43% patients and 86% relapsed patients [12, 13]. Our study is the first attempt to conduct a pan-cancer analysis on MSH2 by using databases of The Cancer Genome Atlas (TCGA), Genotype-Tissue Expression (GTEx), Cancer Cell Line Encyclopedia (CCLE), and others integratedly with relevant factors including gene expression, survival status, genetic alterations, immune infiltration, and associated cellular pathways, and we eventually elucidated MSH2's role in the pathogenesis or the prognosis of cancers. We found that MSH2 expression was positively correlated with the survival prognosis, the immune infiltration, and the tumor load of various tumors, whose correlation with sarcoma (SARC) is more significant.

In the present study, MSH2 expression levels in SARC were significantly associated with genetic differences, tumor immune cell infiltration, and so on, and are likely to be used as target genes for early screening or even therapeutic targets in SARC, which can help improve more than the efficiency of early screening but also the overall survival of SARC patients.

## 2. Materials and Methods

### 2.1. Acquisition of Transcriptional Information

Our analysis to the gene expression patterns in 31 tissues was accomplished with the Genotype-Tissue Expression (GTEx) dataset (https://http://commonfund.nih.gov/GTEx/). Then, the subsequent analysis went along with the information from the CCLE (Cancer Cell Line Encyclopedia) database (https://portals.broadhttp://institute.org/ccle/), which was downloaded for each tumor cell line. The gene expression patterns in 21 tissues were subjected to the analysis according to tissue origin. Then, mRNA information was downloaded from the database of TCGA (https://www.cancer.gov/about-nci/organization/ccg/research/structural-genomics/tcga), which was for an analysis to 31 tumor samples.

The Kruskal-Wallis test was implemented through the R language version 3.6.3 (R Foundation for Statistical Computing, Austria) (https://www.r-project.org/) to determine the expression differences amid organs.

### 2.2. Differential Gene Expression Analysis

We downloaded the datasets of TCGA pan-cancer and GTEx from the UCSC Xena database (https://xena.ucsc.edu/) to figure out the differences in MSH2 expression patterns within our tumor samples and their control normal tissues. First of all, distinction of MSH2 expression patterns within tumor tissues and their control normal tissues in 20 tumor samples was obtained from TCGA database. Given the tiny amount of normal tissue samples in TCGA, we only make an integration of the information about the normal tissues separately from the GTEx database and TCGA tumor tissues, so that our analysis to the gene expression differences in 27 tumors could be performed. Distinction with a threshold of *P* < 0.05 was calculated in R language.

### 2.3. Survival Analysis at the Pan-Cancer Level

To figure out the association amid MSH2 expression patterns and the prognosis of 33 tumors in TCGA cohort, taking into account the possible presence of nontumor mortality factors during follow-up, we performed univariate COX regression analysis by using a threshold of COX (*P* < 0.05) for overall survival (OS), disease-free survival (DFS), disease-specific survival (DSS), disease-free interval (DFI), and progression-free interval (PFI). Summary forest plotting was performed using the R language forest plot package [14]. The tumors with a significant correlation in the regression analysis were selected, and our subjects were divided into two groups of high and low expression on the basis of the median of MSH2 expressions. Our Kaplan-Meier survival analysis was conducted with our R language packages of survival version 3.2.3 and survminer version 0.4.8. A log-rank test with a threshold of *P* < 0.05 was used to calculate the significance of the differences in survival rates.

### 2.4. Relationship between MSH2 Expression Levels and Immunity

Detectable level of tumor-infiltrating lymphocytes (TILs) in tumorous microenvironment suggests an improvement in prognosis and an efficient treatment outcome to different types of cancer [15]. We conducted an investigation to the correlation within MSH2 expression and the level of immune infiltration in different types of tumors. And our exploration on the MSH2's relationship with the immune infiltration level within all the association amid MSH2 expression and tumor-infiltrating lymphocytes in TCGA tumors (B cells, CD4+ T cells, CD8+ T cells, macrophages, neutrophils, and dendritic cells) was carried out by using the Immune-Gene module at the TIMER2 (tumor immune estimation resource, version 2, http://timer.cistrome.org/) online. According to the relevant literature, we chose different study methods for different TILs to improve the accuracy. We used the EPIC method to calculate the relative proportions of B cells, CD4+ T cells, and macrophages of multiple tumors and the QUANTISEQ method to calculate the relative proportions of CD8+ T cells in multiple tumors. After that, we calculated the relative proportions of neutrophils and dendritic cells with the MCPCOUNTER method [16]. When our association analysis came with the QMCPCOUNTER method, we used the function of Purity Adjustment, which means the usage of the partial Spearman's correlation. When it came to the EPIC and QUANTISEQ methods, we affirmed that the parameters of tumor purity and immune infiltration would be negatively correlated; hence, the adjustment to purity became unnecessary [17]. Immune cell infiltration level was estimated with the ESTIMATE method in R language, which comprised the immune microenvironment score as well as the stromal score of 33 tumorous cell samples from TCGA cohort [18]. We determined the association within MSH2 and the immune cell scores above with the Spearman correlation method.

### 2.5. Relationship between MSH2 and Neoantigen, TMB, and MSI

Point mutations, deletion mutations, gene fusions, and so on are the primary reasons of genetic mutations in tumor cells, and most of the mutated genes encode the nascent antigen named neoantigen. New abnormal proteins differ from the ones produced by normal cells. These proteins are enzymatically cleaved to form peptide fragments that are delivered to T cells, which facilitate T cells to be mature activated T cells which could specifically recognize tumor neoantigens and have themselves proliferate [19].

We hence had an estimation to the neoantigen amount in each tumor sample and conducted an analysis on the MSH2 expressions with immune neoantigens in a way of using the Spearman correlation method gene marker correlation [20]. Tumor mutational burden is a parameter usually presented as the somatic mutation amount (nonsynonymous mutations) begetting in an average of 1 Mb bases within the coding region (episomal region) in tumor genomes, which is even straightly shown as the total number of nonsynonymous mutations, as well as the types of mutations which mainly include single-nucleotide variants (SNV) and the insertions/deletions of small fragments' various forms of mutations. Here, we made a calculation separately to the tumor mutational burden (TMB) of each tumor sample and an analysis on the association amid MSH2 expression and TMB with correlation coefficient of Spearman's rank.

Microsatellite instability (MSI) is a term to describe any change in microsatellite length resulting from the insertion or the deletion of repeat units in the particular microsatellite of tumors versus normal tissue. Furthermore, emergence of a new microsatellite allele could be deemed as a genetic phenomenon [21]. We made use of the R data package “PreMSIm” for the prediction on MSI from the gene expression profiles of 33 cancers and commenced an analysis to the relationship within gene expression and MSI by the way of using the Spearman rank correlation coefficient [22].

### 2.6. Mutation Patterns of the MSH2 Gene in TCGA Tumor Samples

Our mutation data were downloaded from TCGA database for 33 malignant tumors, and the changes of the MSH2 gene in these tumors were analyzed. We used the R data package “maftools” to visualize the tumors with the most MSH2 mutations [23].

### 2.7. Gene Enrichment Analysis of Pan-Cancer Patients in TCGA

We first used the STRING website (https://string-db.org/) to query the name “MSH2” using a single protein and “organism” selected from “Homo sapiens.” We then set the following main parameters: minimum required interaction score “low confidence (0.150),” meaning of network edges “confidence,” max number of interactors to show “no more than 50 interactors” in the 1st shell, and active interaction sources “experiments.” Finally, the available MSH2-binding proteins for the experimental assays were obtained.

We used the “Similar Gene Detection” model of Gene Expression Profiling Interactive Analysis 2 (GEPIA2, http://gepia2.cancer-pku.cn/#index) to obtain the top 100 MSH2-related target genes based on data from all TCGA tumors and associated normal tissues. We also performed the Pearson correlation analysis of MSH2 by “correlation analysis” mode of GEPIA2, and the scatter plots were obtained using log2 TPM, *P* value, and the correlation coefficient (*P* value). Value and the correlation coefficient (*R*) have been represented in the graph. In addition, we used the “Gene_Corr” model of TIMER2 to obtain heat map data for the selected genes, including partial correlation (cor) and purity-adjusted Spearman's rank correlation test (*s* rank correlation test).

We combined the two sets of data from the relevant target genes and the binding protein genes for Kyoto Encyclopedia of Genes and Genomes (KEGG) pathway analysis. Briefly, we selected identifier (“OFFICIAL_GENE_ SYMBOL”) and species (“Homo sapiens”) in the DAVID (Database for Annotation, Visualization, and Integrated Discovery) website to obtain functional annotation chart data. The final visualization of the enrichment pathways was obtained through the Sangerbox website (http://sangerbox.com), where we also performed GO (Gene Ontology) enrichment analysis, biological process (BP), cellular component (CC), and molecular function (MF) data visualized as centplots, and two-tailed *P* <0.05 was considered statistically significant.

## 3. Results

### 3.1. Gene Expression Analysis Data

**Figure 1:**
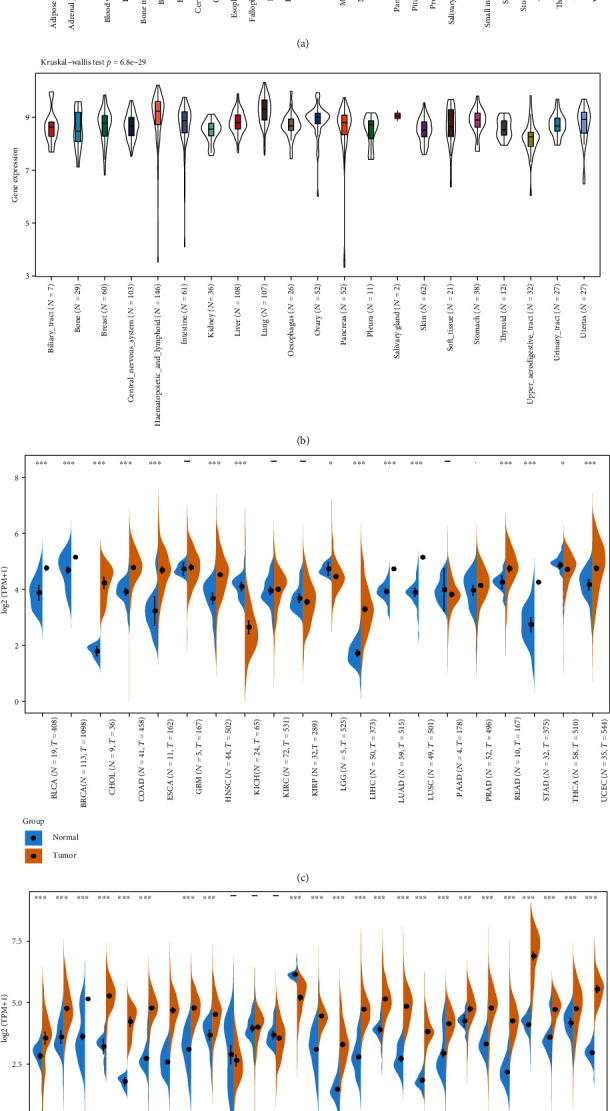
MSH2 expression level in 31 normal tissues across (a) the GTEx dataset and (b) the CCLE database. We downloaded the information of the distinction samples from (c) TCGA database and (d) GTEx datasets on individual gene expression between cancer and paracancer.

We analyzed the differences in gene expression between cancer and paracancer in individual tumor samples obtained from TCGA database, as shown in Figure 1(c). In bladder urothelial carcinoma (BLCA), BRCA, cholangiocarcinoma (CHOL), colon adenocarcinoma (COAD), esophageal carcinoma (ESCA), HNSC, kidney chromophobe (KICH), liver hepatocellular carcinoma (LIHC), lung adenocarcinoma (LUAD), lung squamous cell carcinoma (LUSC), rectum adenocarcinoma (READ), stomach adenocarcinoma (STAD), uterine corpus endometrial carcinoma (UCEC) (*P* value <0.001), LGG, and thyroid carcinoma (THCA) (*P* value <0.05), the tumors in TCGA cohort did not show MSH2 expression levels lower than those of the relevant control normal tissues.

After using normal tissues from the GTEx dataset as controls, we further evaluated the differences of MSH2 expression in adrenocortical carcinoma (ACC), cervical squamous cell carcinoma and endocervical adenocarcinoma (CESC), acute myeloid leukemia (LAML), ovarian serous cystadenocarcinoma (OV), testicular germ cell tumors (TGCT), and uterine carcinosarcoma (UCS). As shown in Figure 1(d), the MSH2 expression levels in ACC, CESC, OV, TGCT, and UCS (*P* value <0.001) were higher than those in the relevant control normal group tissues.

In addition, the Kruskal-Wallis test showed significant differences in MSH2 expression levels among organs (Figures 1(a) and 1(b)), while MSH2 expression levels were significantly higher in bone marrow tissues with a value of log2 (TPM + 1) > 6.

### 3.2. Survival Analysis Data

**Figure 2:**
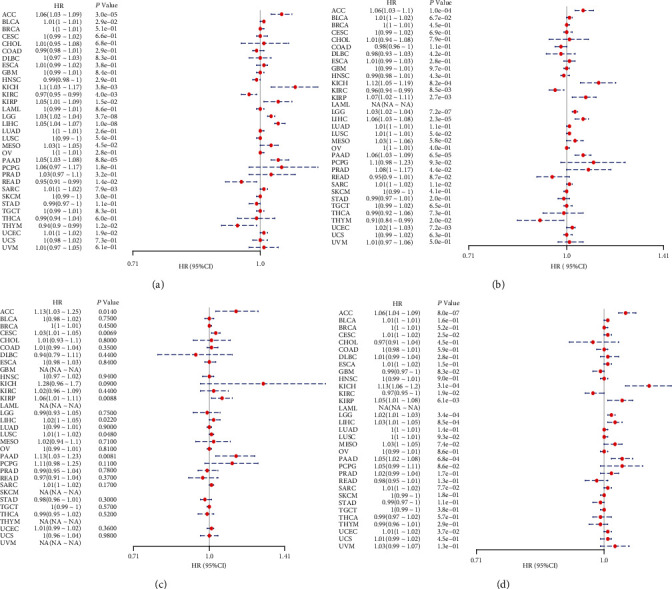
The relationship between expression and OS (a), DSS (b), DFI (c), and PFI (d) in 33 tumors of TCGA. Outcomes of univariate COX regression analysis were shown through the forest plot.

**Figure 3:**
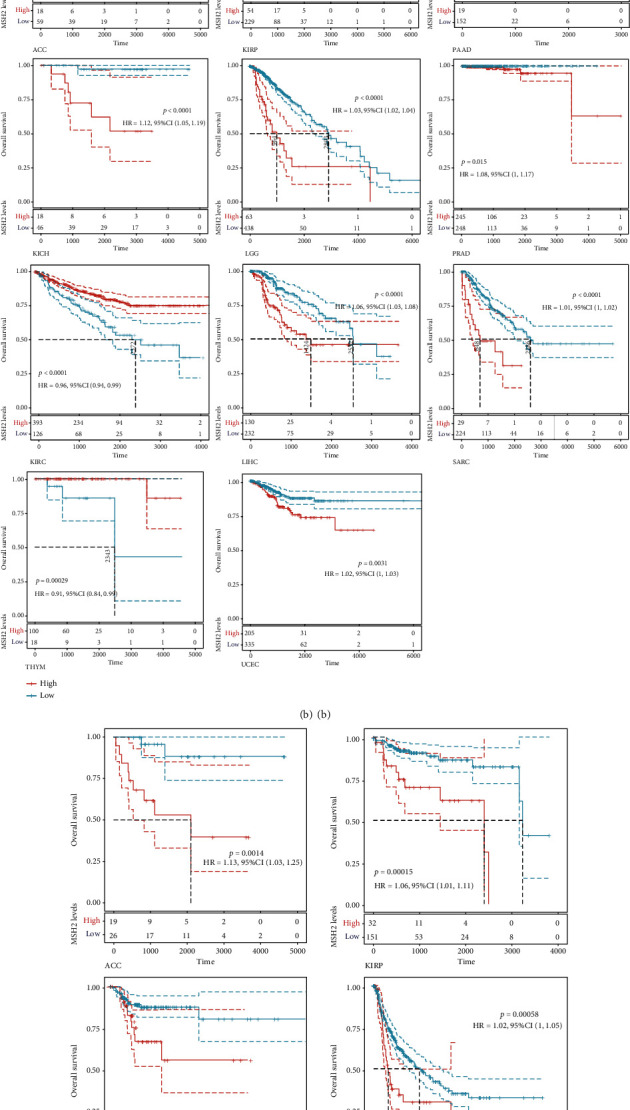
A log-rank test was conducted for the determination on the significance of the overall survival differences (a), DSS differences (b), DFI differences (c), and PFI distinctions (d) with a threshold of *P* < 0.05, whose results were presented by the way of Kaplan-Meier survival curves versus the patients' survival rates of low and high MSH2 expression in tumors.

We investigated the relationship between MSH2 expression levels and survival prognosis in patients with different tumors. We first analyzed the relationship between expression and prognostic OS in 33 tumors of TCGA using gene expression profile data and univariate survival analysis. The forest plots in 33 tumors are shown in Figure 2(a), and among the significant tumors, ACC, BLCA, KICH, kidney renal clear cell carcinoma (KIRC), kidney renal papillary cell carcinoma (KIRP), LGG, LIHC, mesothelioma (MESO), pancreatic adenocarcinoma (PAAD), READ, SARC, thymoma (THYM), and UCEC are selected as prognostic KM curves. We divided cancer cases into high- and low-expression groups according to the median expression level of MSH2 and mainly applied the databases of TCGA and GEO to investigate the relationship between MSH2 expression and prognosis of patients with different tumors. According to Figure 3(a) high expression of MSH2 was associated with poorer prognosis in ACC, BLCA, KICH, KIRP, LGG, LIHC, MESO, PAAD, SARC, and UCEC, while low expression of MSH2 was associated with poorer prognosis in KIRC, READ, and THYM.

Also, considering the possibility of non-tumor-related deaths during follow-up, we analyzed the relationship between gene expression and DSS in 33 tumors of TCGA cohort (Figure 2(b)); among the significant tumors, ACC, KICH, KIRC, KIRP, LGG, LIHC, PAAD, PRAD, SARC, THYM, and UCEC are selected as prognostic KM curves. Cancer cases were divided into high- and low-expression groups according to the median expression level of MSH2 for prognostic KM curves. As shown in Figure 3(b) MSH2 was expressed in ACC, KICH, KIRP, LGG, LIHC, PAAD, PRAD, SARC, and UCEC in which high expression levels were significantly associated with their poorer DSS, while in KIRC and THYM, low MSH2 expression levels were associated with poorer DSS.

**Figure 4:**
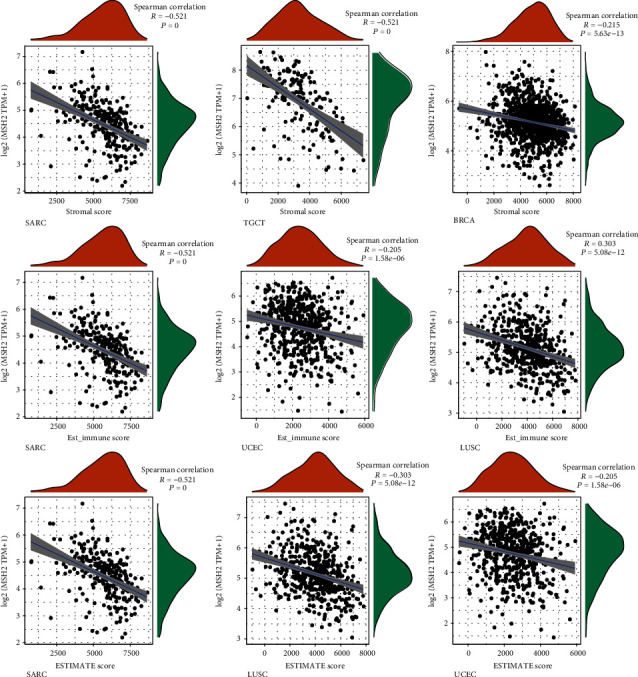
Correlation of MSH2 expression with the stromal score, immune score, and ESTIMATE score in SARC, LUSC, UCEC, TGCT, and BRCA.

We further analyzed the relationship between gene expression and DFI (Figure 2(c)) and PFI (Figure 2(d)) in the 33 tumors of TCGA cohort. Significant tumors (ACC, CESC, KIRP, LIHC, LUSC, PAAD and ACC, CESC, KICH, KIRC, KIRP, LGG, LIHC, PAAD, and UCEC) were selected in DFI and PFI survival analysis, and cancer cases were divided into high- and low-expression groups according to MSH2 expression levels for prognostic KM curves. As shown in Figure 3(c), in the DFI survival analysis, high expression of MSH2 was all associated with poorer prognosis in ACC, CESC, KIRP, LIHC, LUSC, and PAAD. As shown in Figure 4 (d), in the PFI survival analysis, high expression of MSH2 was associated with poorer prognosis in ACC, CESC, KICH, KIRP, LGG, LIHC, PAAD, and UCEC. And low expression of MSH2 was associated with a worse prognosis for KIRC patients.

### 3.3. Relationship between Gene Expression and Immunity in Individual Tumors

Tumor-infiltrating lymphocytes are independent predictors of anterior lymph node status and survival in cancer [24]. We investigated whether this gene expression correlated with the level of immune infiltration in different types of cancers.

The results showed that MSH2 expression levels were significantly correlated with the level of B cell infiltration in 18 cancers, CD4+ T cell infiltration in 23 cancers, CD8+ T cells in 10 cancers, macrophages in 12 cancers, neutrophils in 26 cancers, and dendritic cells in 12 cancers. The three most significantly correlated tumors in each immune cell were selected. B cell infiltration levels were significantly correlated with MSH2 expression levels in LGG, KIRP, and PRAD. CD4+ T cell infiltration level was significantly correlated with MSH2 expression levels in THCA, HNSC, and KIRC. CD8+ T cell infiltration level was significantly correlated with MSH2 expression levels in THYM, LIHC, and SARC. Macrophage infiltration levels were significantly correlated with MSH2 expression levels in LIHC, glioblastoma multiforme (GBM), and SARC. Neutrophil infiltration levels were significantly correlated with MSH2 expression levels in THYM, KIRC, and PRAD. Dendritic cell infiltration levels were significantly correlated with MSH2 expression levels in BRCA, HNSC, and LIHC.

An increasing number of reports suggest that the tumor immune microenvironment has an important role in tumor development [25]. We observed the relationship between gene expression and the immune score, stromal score, and ESTIMATE score in 33 tumors and selected the three tumors with the most significant relationship among each score as shown in Figure 4. The results showed that the expression levels of MSH2 in SARC, TGCT, and BRCA were significantly and negatively correlated with the stromal score. The MSH2 gene expression levels in SARC, UCEC, and LUSC were significantly and positively correlated with the immune score. In SARC, LUSC, and UCEC, MSH2 gene expression levels were significantly and positively correlated with the ESTIMATE score.

**Figure 5:**
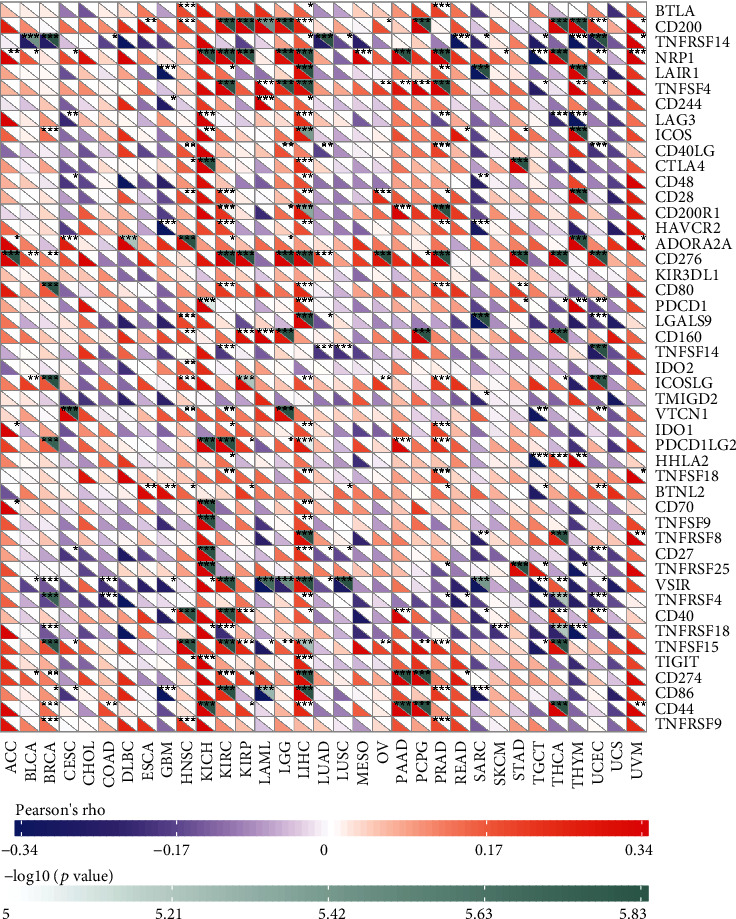
MSH2's relationship with immune checkpoint gene expression was presented in a way of a heat map. The horizontal coordinates indicate the 33 selected tumors, and the vertical coordinates indicate the relevant immune checkpoints, where ∗ indicates correlation (*P* < 0.05), ∗∗ indicates high correlation (*P* < 0.01), and ∗∗∗ indicates significant correlation (*P* < 0.001).

Under normal conditions, immune cells can recognize and remove tumor cells from the tumor microenvironment [26]. Tumor immunotherapy approaches control and eliminate immune cells by restarting and maintaining the tumor immune cycle as a means to repair the normal antitumor immune response in the body. Immune checkpoint genes include monoclonal antibody-based immune checkpoint inhibitors, therapeutic antibodies, cancer vaccines, cell therapy, and small-molecule inhibitors [27]. As shown in Figure 5, the horizontal coordinates indicate the 33 selected tumors and the vertical coordinates indicate the relevant immune checkpoints. We found that the expression of MSH2 was positively correlated with the expression levels of immune checkpoint genes in KICH, KIRC, and LICH, while the expression of MSH2 was negatively correlated with the expression levels of immune checkpoint genes in SARC.

### 3.4. Relationship between Gene Expression and Immune Neoantigens, TMB, and Microsatellite Instability

**Figure 6:**
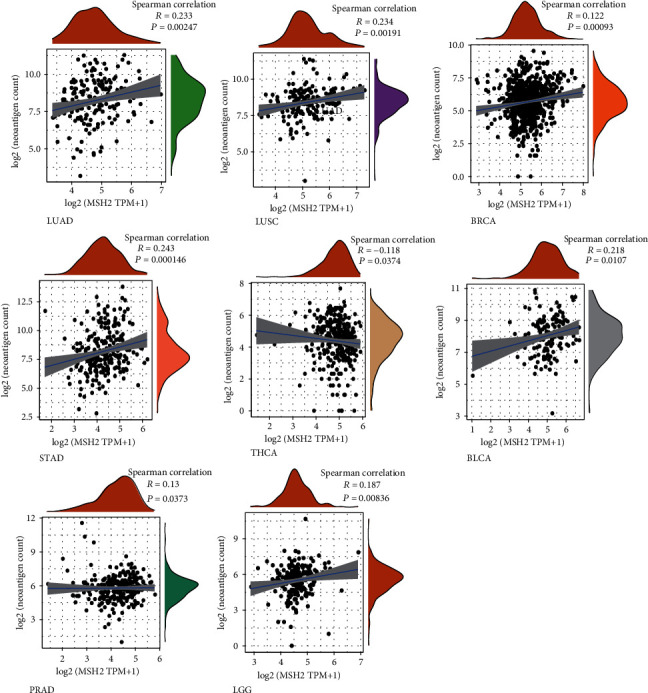
MSH2's correlation with neoantigens. Expression of MSH2 was positively correlated with the immune neoantigens' amount in LUAD, LUSC, BRCA, STAD, THCA, BLCA, PRAD, and LGG.

The immune activity of tumor neoantigens and neoantigen vaccines can be designed and synthesized according to the mutation of tumor cells and immunized to patients to achieve therapeutic effects [28]. Here, we counted the number of neoantigens in each tumor sample separately to analyze the relationship between MSH2 expression and the number of antigens. As shown in Figure 6, the expression levels of MSH2 in LUAD, LUSC, BRCA, STAD, THCA, BLCA, PRAD, and LGG were found positively correlated with the number of immune neoantigens.

**Figure 7:**
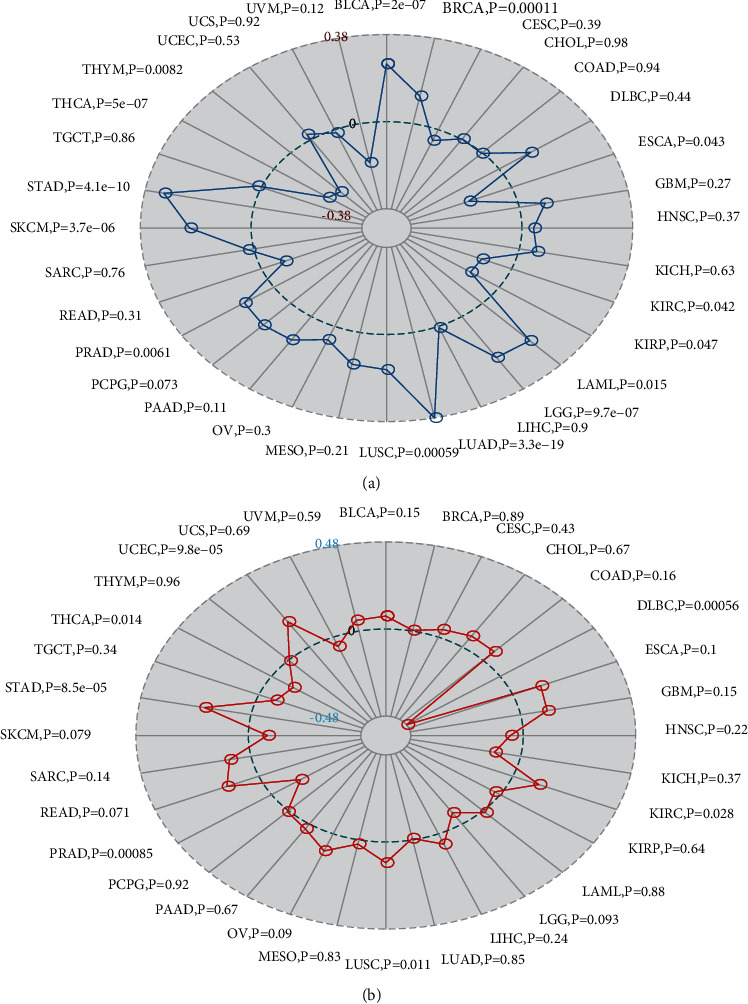
MSH2's correlation separately with TMB (a) and microsatellite instability (b).

TMB is used to reflect the number of mutations contained in tumor cells and is a quantifiable biomarker. Here, we counted TMB for each tumor sample separately using Spearman's rank correlation coefficient and analyzed the relationship between gene expression and TMB as shown in Figure 7(a). MSH2 gene expression level results such as BLCA, BRCA, LAML, LGG, LUAD, LUSC, PRAD, skin cutaneous melanoma (SKCM), and STAD were significantly and positively correlated with TMB, while ESCA, KIRC, KIRP, THCA, and THYM showed a negative correlation between MSH2 gene expression levels and TMB.

We analyzed the correlation between gene expression and MSI using the Spearman rank correlation coefficient as shown in Figure 7(b). The results were as follows: MSH2 gene expression levels in KIRC, LUSC, STAD, and UCEC were positively correlated with MSI, while lymphoid neoplasm diffuse large B cell lymphoma (DLBC), PRAD, and THCA showed a negative correlation between MSH2 gene expression levels and MSI.

### 3.5. Mutation Patterns of Genes in Individual Tumor Samples

**Figure 8:**
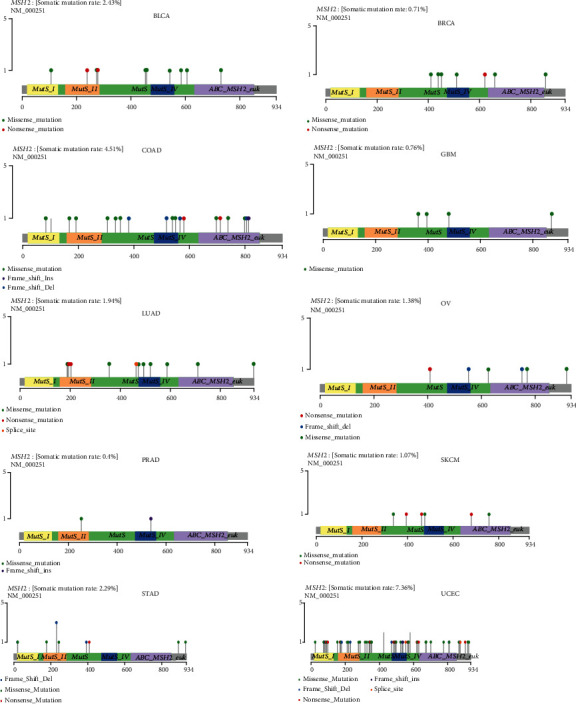
MSH2 gene mutation patterns in several tumors.

We obtained mutation data from TCGA database for 33 tumors and analyzed the mutations of MSH2 in these tumors. As shown in Figure 8, MSH2 was observed to mutate in BLCA, BRCA, COAD, GBM, LUAD, OV, PRAD, SKCM, STAD, and UCEC. The top three tumors with the highest MSH2 mutation rate were UCEC (rate = 7.36%), COAD (rate = 4.51%), and BRCA (rate = 2.43%).

### 3.6. Enrichment Analysis of MSH2-Related Partners

**Figure 9:**
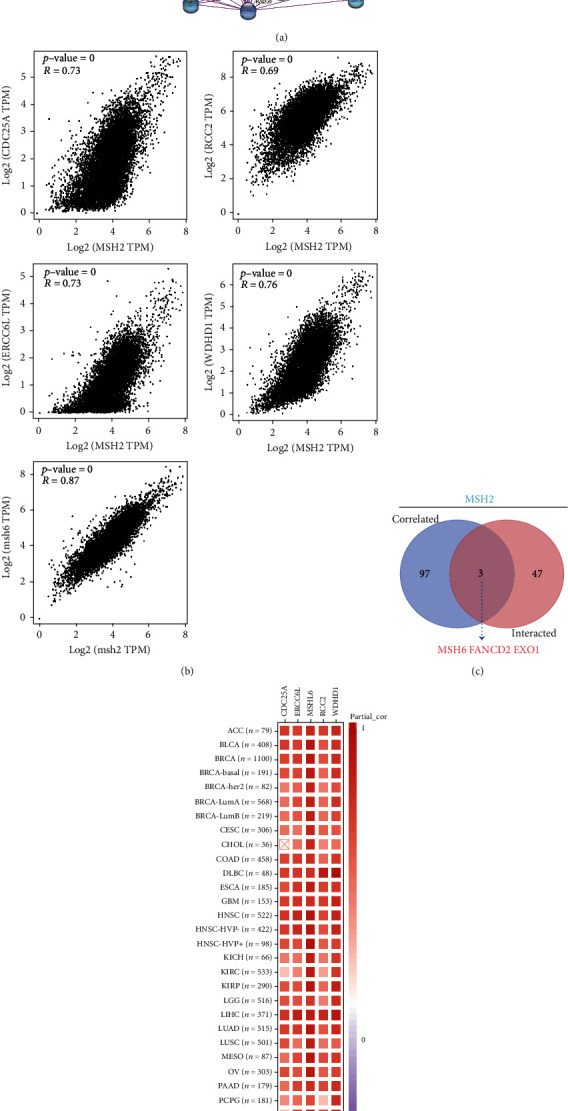
Enrichment analysis of MSH2-binding proteins and MSH2 expression-related genes. (a) We obtained the result of the experimentally available determination to the MSH2-binding proteins with the STRING tool. (b) We obtain 100 of the genes with the closest association with MSH2 expression. MSH2 expression levels were most positively correlated with MSH6, WDHD1, CDC25A, ERCC6L, and RCC2. (c) The intersection of the above two datasets showed three common genes, MSH6, FANCD2, and EXO1. (d) Information of the corresponding heat map also presented us a positive relationship amid MSH2 and the five genes above in most cancer types.

To further understand the molecular mechanisms of MSH2 in tumorigenesis, we screened for MSH2-binding proteins and MSH2 expression-related genes for a series of enrichment analyses. Based on the STRING website, we obtained a total of 50 MSH2-binding proteins supported by experimental evidence. The network diagram of the interactions of these proteins is shown in Figure 9(a). Using the GEPIA2 website, we combined the expression data of all tumor and normal tissues in TCGA to obtain the top 100 genes associated with MSH2 expression. As shown in Figure 9(b), MSH2 expression levels were positively correlated with MSH6, WDHD1, CDC25A, ERCC6L, and RCC2 (all *P* < 0.001). The corresponding heat map data also showed a positive correlation between MSH2 and the above five genes in most cancer types (Figure 9(d)). The intersection of the above two datasets showed three common genes, MSH6, FANCD2, and EXO1 (Figure 9(c)).

**Figure 10:**
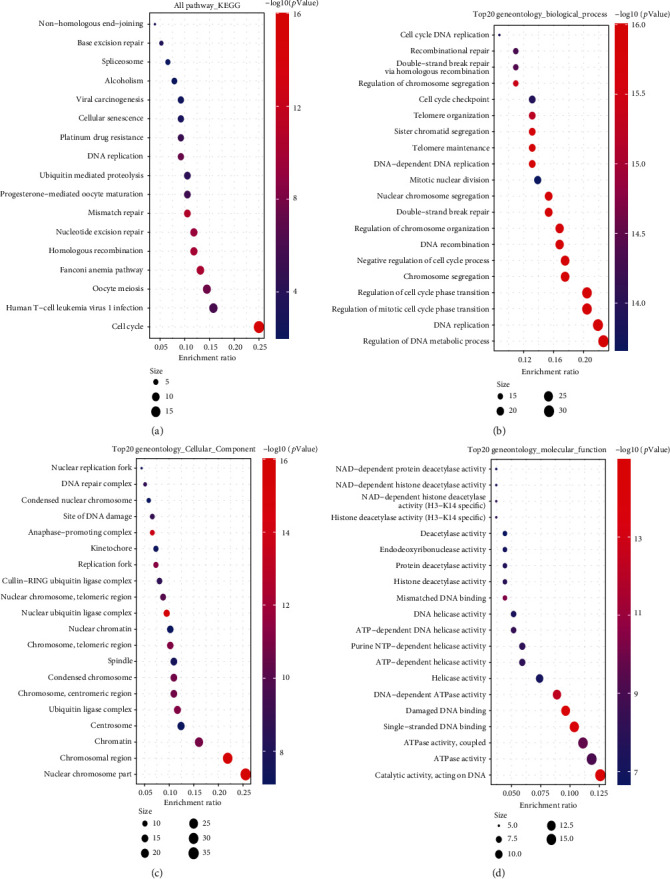
KEGG pathway GO enrichment analysis was performed on the basis of MSH2-binding and interacted genes. (a) KEGG pathway analysis. (b) Biological process, (c) cellular component, and (d) molecular function information of GO analysis was presented as a bubble plot.

We combined these two datasets to perform KEGG and GO enrichment analysis, as shown in Figure 10, where the KEGG data suggest that the “cell cycle” may be involved in the influence of MSH2 on tumor pathogenesis, and the GO enrichment analysis data further suggest that the molecular mechanisms of these genes are mostly related to DNA metabolic pathways or chromosomal cell biology, such as “regulation of DNA metabolic process” and “DNA replication.”

## 4. Discussion and Conclusions

China is the country with the most population worldwide; with the rising amount of its aging population, the burden of cancer in China comes to be severe [29]. Meanwhile, since the novel coronavirus pandemic in 2019, studies have shown that cancer patients in a state of systemic immunosuppression are considered highly vulnerable to the COVID-19 epidemic [30, 31].

We made a comprehensive examination on the MSH2 gene with a total of 33 different tumors in TCGA cohort based on data from TCGA, CCLE, UCSC Xena, and GTEx databases, as well as gene expression, gene variants, methylation, immune infiltration, and enrichment analysis [32]. Then, it turned out that expression of MSH2 was significantly related to prognosis and immunity in several different tumors. Therefore, we could assume that MSH2 might be a screening indicator and a possible factor for multiple tumors in the future.

We observed differences in MSH2 expression within cancers and its control normal tissues. Moreover, MSH2 was significantly more highly expressed in sarcoma, hepatocellular carcinoma, lung cancer, bile duct cancer, prostate cancer, gastric cancer, thyroid cancer, and common genital tumors versus normal tissues, with MSH2 expression being significantly higher in bone marrow tissues. The deletion of MSH2 protein was associated with the inactivation of MSH2, high mutation, and high tumor-infiltrating lymphocyte density in high-grade primary tumors [33]. Because MSH2 protein directs the production of proteins that modulates DNA repair, the MSH2 gene was also considered an oncogene in past studies [34], which is consistent with our analysis that high MSH2 expression was associated with OS in ACC, BLCA, and KICH patients. KIRP, LGG, LIHC, MESO, PAAD, SARC, and UCEC were associated with poorer prognosis in OS, and only KIRC and READ were associated with better prognosis in our analysis. Based on previous clinical studies, MSH2 plays different roles in different cancers, and high MSH2 expression in early-stage lung cancer is significantly associated with poorer prognosis [35], and high expression of MSH2 in NSCLC could be used as a prognostic indicator for prolonged survival [36]. This may be because the action of MSH2 protein depends on the regulation of tumor microenvironment; for example, both class IIb HDACsh and MSH2 may influence tumor pathogenesis through the cell cycle, and the deacetylation of MSH2 by HDAC10 may lead to DNA mismatch repair activity [37].

Our analysis to MSH2 expressions and immunity showed that the MSH2 expression in SARC showed a negative correlation with B cells, CD4+ T cells, CD8+ T cells, macrophages, neutrophils, and dendritic cells; it was also alike in the immune score, stromal score, and ESTIMATE score of ESTIMATE analysis. Progress of tumor development is complex, where the interplays within the cancer cells, microenvironment, and immune system hold impacts on tumorigenesis and progression [38]. Immune cells, by eliminating pathogens, have an important secondary role in maintaining tissue integrity and normal function in different states of homeostasis, infection, and noninfectious disturbances of the body and have an impact on the clinical outcome of tumors [39]. In addition, it has been shown that high or moderate immune scores in SARC can lead to better DFS or OS. Therefore, fortified MSH2 expression associated with worse prognosis in SARC patients may be related to the fact that MSH2 expression suppresses the infiltration of immune cells in the tumor microenvironment and decreases immune scores. Besides that, the MSH2 expressions in SARC presented a significantly negative correlation with most immune check genes, especially LGALS9 and VSIR. Immune checkpoints are various immunosuppressive pathways that hold the balance of self-tolerance, regulating the duration as well as the magnitude of immune responses in the physical state [40]. Immune checkpoint blockade can reduce immune escape of tumor cells and limit tumor growth.

It was reported that the abnormal expression of MSH2 in osteosarcoma cells has been proven a possible sign of drug resistance to chemotherapeutic drugs [41], and case reports have revealed the relationship between MSH2 variants and the development of osteosarcoma, and the accumulation of genetic damage due to MSH2 variants may contribute to the development of osteosarcoma [42]. In a related study on osteosarcoma tissue microarray, local expressions of MSH6 and MSH2/6 were significantly related to shorter survival time, especially in chemotherapy-naive patients and patients with metastatic tumors [43], which is consistent with our findings. However, the study is limited in public databases, and further investigation in MSH2 expression affecting the diagnosis and prognosis of different cancer types is needed. In particular, a potential role of MSH2 indicates the SARC and contributes to the immunotherapy of SARC. This inspirits the future research on verification of the specific role of MSH2 expression on sarcoma and exploring the mechanism of it. In conclusion, the present study firstly conducted the pan-cancer analysis on MSH2 in gene expression, survival status, genetic alterations, immune infiltration, and associated cellular pathways. The study revealed that MSH2 may be an ideal prognostic indicator for SARC as well as a therapeutic target for immunotherapy in the clinical setting to improve patient prognosis and increase survival rates.

## Data Availability

All data generated or analyzed during this study are included in this published article.

## Additional Points

*Highlights*. (1 )The expression of MSH2 is found high in sarcoma and low in normal tissues. (2) High MSH2 in tumors accounts for unfavorable OS in SARC patients. (3) MSH2 may be used as a prognostic indicator for SARC or a therapeutic target for immunotherapy.

## References

[1] de Wind N., Dekker M., Claij N. (1999). HNPCC-like cancer predisposition in mice through simultaneous loss of Msh3 and Msh6 mismatch-repair protein functions. *Nature Genetics*.

[2] Carethers J. M. (2019). High predictability for identifying lynch syndrome via microsatellite instability testing or immunohistochemistry in all lynch-associated tumor types. *Translational Cancer Research*.

[3] Kinsella T. J., Gurkan-Cavusoglu E., Du W., Loparo K. A. (2011). Integration of principles of systems biology and radiation biology: toward development of in silico models to optimize IUdR-mediated radiosensitization of DNA mismatch repair deficient damage tolerant human cancers. *Frontiers in Oncology*.

[4] Dietlein F., Reinhardt H. C. (2014). Molecular pathways: exploiting tumor-specific molecular defects in DNA repair pathways for precision cancer therapy. *Clinical cancer research : an official journal of the American Association for Cancer Research*.

[5] Kastan M. (2008). DNA damage responses: mechanisms and roles in human Disease. *Molecular Cancer Research : MCR*.

[6] Elliott B., Jasin M. (2001). Repair of double-strand breaks by homologous recombination in mismatch repair-defective mammalian cells. *Molecular and Cellular Biology*.

[7] Villemure J. F., Abaji C., Cousineau I., Belmaaza A. (2003). MSH2-deficient human cells exhibit a defect in the accurate termination of homology-directed repair of DNA double-strand breaks. *Cancer Research*.

[8] Mellon I., Rajpal D. K., Koi M., Boland C. R., Champe G. N. (1996). Transcription-coupled repair deficiency and mutations in human mismatch repair genes. *Science*.

[9] Pitsikas P., Lee D., Rainbow A. J. (2007). Reduced host cell reactivation of oxidative DNA damage in human cells deficient in the mismatch repair gene hMSH2. *Mutagenesis*.

[10] Janavicius R., Elsakov P. (2012). Novel germline MSH2 mutation in lynch syndrome patient surviving multiple cancers. *Hereditary Cancer in Clinical Practice*.

[11] Wang Y. C., Lu Y. P., Tseng R. C. (2003). Inactivation of hMLH1 and hMSH2 by promoter methylation in primary non-small cell lung tumors and matched sputum samples. *The Journal of Clinical Investigation*.

[12] Wang C. X., Wang X., Liu H. B., Zhou Z. H. (2014). Aberrant DNA methylation and epigenetic inactivation of hMSH2 decrease overall survival of acute lymphoblastic leukemia patients via modulating cell cycle and apoptosis. *Asian Pacific Journal of Cancer Prevention : APJCP*.

[13] Diouf B., Cheng Q., Krynetskaia N. F. (2011). Somatic deletions of genes regulating MSH2 protein stability cause DNA mismatch repair deficiency and drug resistance in human leukemia cells. *Nature Medicine*.

[14] Ryan C. J., Smith M. R., de Bono J. S. (2013). Abiraterone in metastatic prostate cancer without previous chemotherapy. *The New England Journal of Medicine*.

[15] de Ruiter E. J., Ooft M. L., Devriese L. A., Willems S. M. (2017). The prognostic role of tumor infiltrating T-lymphocytes in squamous cell carcinoma of the head and neck: a systematic review and meta-analysis. *Oncoimmunology*.

[16] Sturm G., Finotello F., Petitprez F. (2019). Comprehensive evaluation of transcriptome-based cell-type quantification methods for immuno-oncology. *Bioinformatics Oxford, England*.

[17] Li T., Fu J., Zeng Z. (2020). TIMER2.0 for analysis of tumor-infiltrating immune cells. *Nucleic Acids Research*.

[18] Yoshihara K., Shahmoradgoli M., Martínez E. (2013). Inferring tumour purity and stromal and immune cell admixture from expression data. *Nature Communications*.

[19] Srivastava R. M., Purohit T. A., Chan T. A. (2020). Diverse neoantigens and the development of cancer therapies. *Seminars in Radiation Oncology*.

[20] Boegel S., Castle J. C., Kodysh J., O'Donnell T., Rubinsteyn A. (2019). Bioinformatic methods for cancer neoantigen prediction. *Progress in Molecular Biology and Translational Science*.

[21] Bonneville R., Krook M. A., Kautto E. A. (2017). Landscape of microsatellite instability across 39 cancer types. *JCO Precision Oncology*.

[22] Li L., Feng Q., Wang X. (2020). PreMSIm: an R package for predicting microsatellite instability from the expression profiling of a gene panel in cancer. *Computational and Structural Biotechnology Journal*.

[23] Yin L., Xiao L., Gao Y. (2020). Comparative bioinformatical analysis of pancreatic head cancer and pancreatic body/tail cancer. *Medical Oncology*.

[24] Yusuf M., Gaskins J., Mandish S. (2020). Tumor infiltrating lymphocyte grade in Merkel cell carcinoma: relationships with clinical factors and independent prognostic value. *Acta oncologica Stockholm, Sweden*.

[25] Dai D., Liu L., Huang H. (2021). Nomograms to predict the density of tumor-infiltrating lymphocytes in patients with high-grade serous ovarian cancer. *Frontiers in Oncology*.

[26] Massari F., Santoni M., Ciccarese C., Santini D. (2015). The immunocheckpoints in modern oncology: the next 15 years. *Expert Opinion on Biological Therapy*.

[27] Danilova L., Ho W. J., Zhu Q. (2019). Programmed cell death ligand-1 (PD-L1) and CD8 expression profiling identify an immunologic subtype of pancreatic ductal adenocarcinomas with favorable survival. *Cancer Immunology Research*.

[28] Li L., Goedegebuure S. P., Gillanders W. E. (2017). Preclinical and clinical development of neoantigen vaccines. *Annals of Oncology*.

[29] Sun D., Li H., Cao M. (2020). Cancer burden in China: trends, risk factors and prevention. *Cancer Biology & Medicine*.

[30] Yang S., Zhang Y., Cai J., Wang Z. (2020). Clinical characteristics of COVID-19 after gynecologic oncology surgery in three women: a retrospective review of medical records. *The Oncologist*.

[31] Song J., Han J., Liu F. (2020). Systematic analysis of coronavirus disease 2019 (COVID-19) receptor ACE2 in malignant tumors: pan-cancer analysis. *Frontiers in Molecular Biosciences*.

[32] Yi Y., Zhang Q., Shen Y. (2022). System analysis of adaptor-related protein complex 1 subunit mu 2 (AP1M2) on malignant tumors: a pan-cancer analysis. *Journal of Oncology*.

[33] Guedes L. B., Antonarakis E. S., Schweizer M. T. (2017). MSH2 loss in primary prostate cancer. *Clinical Cancer Research*.

[34] Fang M., Pak M. L., Chamberlain L., Xing W., Yu H., Green M. R. (2015). The CREB coactivator CRTC2 is a lymphoma tumor suppressor that preserves genome integrity through transcription of DNA mismatch repair genes. *Cell Reports*.

[35] Levallet G., Dubois F., Fouret P. (2017). MSH2/BRCA1 expression as a DNA-repair signature predicting survival in early-stage lung cancer patients from the IFCT-0002 phase 3 trial. *Oncotarget*.

[36] Bonomi M., Pilotto S., Milella M. (2011). Adjuvant chemotherapy for resected non-small-cell lung cancer: future perspectives for clinical research. *Journal of Experimental & Clinical Cancer Research : Cr*.

[37] Li Y., Seto E. (2016). HDACs and HDAC inhibitors in cancer development and therapy. *Cold Spring Harbor Perspectives in Medicine*.

[38] Chen D. S., Mellman I. (2017). Elements of cancer immunity and the cancer-immune set point. *Nature*.

[39] Sui S., An X., Xu C. (2020). An immune cell infiltration-based immune score model predicts prognosis and chemotherapy effects in breast cancer. *Theranostics*.

[40] Pardoll D. M. (2012). The blockade of immune checkpoints in cancer immunotherapy. *Nature Reviews. Cancer*.

[41] Fujii H., Honoki K., Tsujiuchi T., Kido A., Yoshitani K., Takakura Y. (2009). Sphere-forming stem-like cell populations with drug resistance in human sarcoma cell lines. *International Journal of Oncology*.

[42] Ahmed H., Salama A., Salem S. E., Bahnassy A. A. (2012). A case of synchronous double primary breast carcinoma and osteosarcoma: mismatch repair genes mutations as a possible cause for multiple early onset malignant tumors. *The American Journal of Case Reports*.

[43] Jentzsch T., Robl B., Husmann M., Bode-Lesniewska B., Fuchs B. (2014). Expression of MSH2 and MSH6 on a tissue microarray in patients with osteosarcoma. *Anticancer Research*.

