# Correction to “A Pan‐Cancer Analysis on the Systematic Correlation of MutS Homolog 2 (MSH2) to a Malignant Tumor”

**DOI:** 10.1155/jo/9756191

**Published:** 2026-09-30

H. Yao, Z. Cao, H. Yong, et al., “A Pan‐Cancer Analysis on the Systematic Correlation of MutS Homolog 2 (MSH2) to a Malignant Tumor,” *Journal of Oncology* 2022, no. 1 (2022): 1–22, https://doi.org/10.1155/2022/9175402.

In the article titled “A Pan‐Cancer Analysis on the Systematic Correlation of MutS Homolog 2 (MSH2) to a Malignant Tumor,” there were multiple errors the article’s text and figures. These errors are shown and corrected below:

Error in Figure 4:

Some scatter plots in Figure 4 were unintentionally duplicated as a result of an error in the code used for visualization, resulting in repetition. The correct version of Figure 4 is shown below.

**FIGURE 4:**
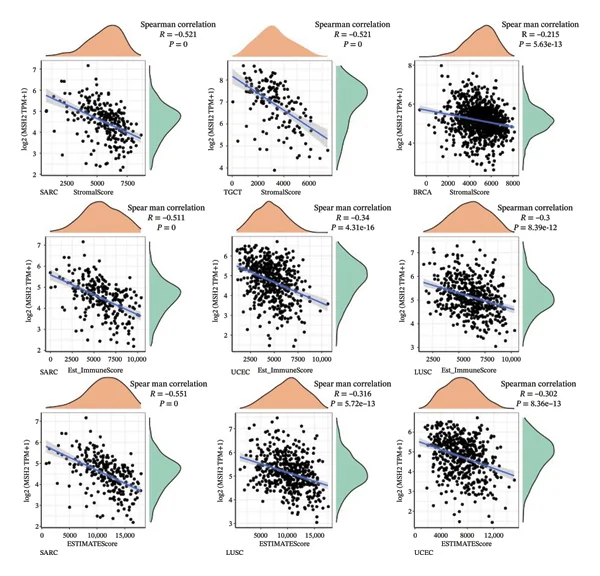
Correlation of MSH2 expression with the stromal score, immune score, and ESTIMATE score in SARC, LUSC, UCEC, TGCT, and BRCA.

Error in Section 3.2 *Survival Analysis Data*, page 10:

“As shown in Figure 4(d), in the PFI survival analysis, high expression of MSH2 was associated with poorer prognosis in ACC, CESC, KICH, KIRP, LGG, LIHC, PAAD, and UCEC.”

should read as follows:

“As shown in Figure 3(d), in the PFI survival analysis, high expression of MSH2 was associated with poorer prognosis in ACC, CESC, KICH, KIRP, LGG, LIHC, PAAD, and UCEC.”

Error in Section 3.3 *Relationship between Gene Expression and Immunity in Individual Tumors*, page 11:

“The MSH2 gene expression levels in SARC, UCEC, and LUSC were significantly and positively correlated with the immune score. In SARC, LUSC, and UCEC, MSH2 gene expression levels were significantly and positively correlated with the ESTIMATE score.”

should read as follows:

“The MSH2 gene expression levels in SARC, UCEC, and LUSC were significantly and negatively correlated with the immune score. In SARC, LUSC, and UCEC, MSH2 gene expression levels were significantly and negatively correlated with the ESTIMATE score.”

Error in Section 3.4 *Relationship between Gene Expression and Immune Neoantigens*, *TMB, and Microsatellite Instability*, page 12:

“As shown in Figure 6, the expression levels of MSH2 in LUAD, LUSC, BRCA, STAD, THCA, BLCA, PRAD, and LGG were found positively correlated with the number of immune neoantigens.”

should read as follows:

“As shown in Figure 6, the expression levels of MSH2 in LUAD, LUSC, BRCA, STAD, BLCA, PRAD, and LGG were found positively correlated with the number of immune neoantigens.”

Error in the legend of Figure 6, page 13:

“MSH2’s correlation with neoantigens. Expression of MSH2 was positively correlated with the immune neoantigens’ amount in LUAD, LUSC, BRCA, STAD, THCA, BLCA, PRAD, and LGG.”

should read as follows:

“MSH2’s correlation with neoantigens. Expression of MSH2 was positively correlated with the immune neoantigens’ amount in LUAD, LUSC, BRCA, STAD, BLCA, PRAD, and LGG.”

Error in Section 3.5 *Mutation Patterns of Genes in Individual Tumor Samples*, page 13:

“The top three tumors with the highest MSH2 mutation rate were UCEC (rate = 7:36%), COAD (rate = 4:51%), and BRCA (rate = 2:43%).”

should read as follows:

“The top three tumors with the highest MSH2 mutation rate were UCEC (rate = 7:36%), COAD (rate = 4:51%), and BLCA (rate = 2:43%).”

We apologize for these errors.

